# miR-30d inhibits cell biological progression of Ewing's sarcoma by suppressing the MEK/ERK and PI3K/Akt pathways *in vitro*

**DOI:** 10.3892/ol.2020.12394

**Published:** 2020-12-18

**Authors:** Conglin Ye, Xiaolong Yu, Xuqiang Liu, Min Dai, Bin Zhang

Oncol Lett 15: 4390-4396, 2018; DOI: 10.3892/ol.2018.7900

Subsequently to the publication of the above article, an interested reader drew to the authors' attention that, in Fig. 1D on p. 4392, in the 0 h row the ‘Scramble’ and ‘mimic’ panels appeared to feature overlapping data.

After having re-examined their data, the authors realize that the Figure had been assembled incorrectly, and they were able to identify the correct image for the ‘Scramble’ panel. The corrected version of Fig. 1, including the corrected data for Fig. 1D, is shown below. Note that the error made in this Figure did not affect the results or the conclusions reported in this paper, and all the authors agree to this Corrigendum. The authors thank the Editor of *Oncology Letters* for presenting them with the opportunity to publish this Corrigendum, and apologize to the Editor and to the readership of the Journal for any inconvenience caused.

## Figures and Tables

**Figure 1.**
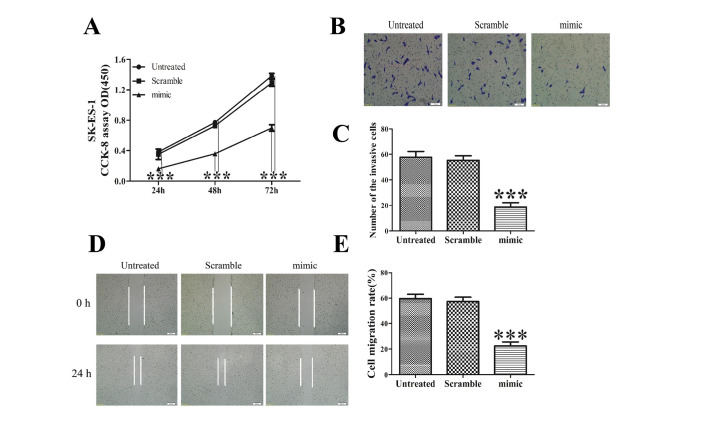
Increased miR-30d inhibits proliferation, migration and invasion of SK-ES-1 cells. (A) CCK-8 assay revealed that ectopic miR-30d significantly suppressed cell growth in SK-ES-1 cells (***P<0.001, vs. untreated). Transwell assay (B) images (magnification, ×100) and (C) quantification demonstrated that invasive potential was significantly reduced in the miR-30d mimic group relative to the untreated group (***P<0.001, vs. untreated). Wound healing assay (D) images (magnification, ×40) and (E) quantification showed that the overexpression of miR-30d markedly repressed the migration of SK-ES-1 cells compared with the control group (***P<0.001, vs. untreated). miR, microRNA; CCK-8, Cell Counting Kit-8.

